# Development of a Multi-Criteria Design Optimization Methodology for Automotive Plastics Parts

**DOI:** 10.3390/polym14010156

**Published:** 2021-12-31

**Authors:** Victor J. Romero, Alberto Sanchez-Lite, Gerard Liraut

**Affiliations:** 1 Department of Materials Science and Metallurgical Engineering, Graphic Expression in Engineering, Cartographic Engineering, Geodesy and Photogrammetry, Mechanical Engineering and Manufacturing Engineering, Universidad de Valladolid, 47011 Valladolid, Spain; victor.romero@uva.es; 2 Société Française des Ingénieurs des Plastiques, SFIP, Le Diamant A., La Défense CEDEX, 92909 Paris, France; gerard.liraut@sfip-plastic.org

**Keywords:** design, engineering, design optimization, multi-criteria, automotive, plastic, polymer, sustainability, design to cost, eco design, plastic injection

## Abstract

The plastic industry is undergoing drastic changes, due to the customer sustainability perception of plastics, and the eruption of new processes (such 3D printing) and materials (such as renewably sourced resins). To enable a fast transition to high-quality, sustainable plastic applications, a specific methodology could be a key competitive advantage. This novel methodology is focused on improving the objectivity and efficiency of plastic production and the design review process. It is applicable to discrete optimization events in any product lifecycle milestone, from concept design to serial production stages. The methodology includes a natural way to capture plastic-related knowledge and trends, oriented towards building a dynamic “interaction matrix”, with a list of potential optimizations and their positive or negative impacts in a comprehensive set of multi-criteria evaluations. With an innovative approach, the matrix allows the possibility to incorporate a business strategy, which could be different at every lifecycle stage. The business strategy is translated from the common “verbal” definition into a quantitative set of “Target and Restrictions”, making it possible to detect and prioritize the best potential design optimization changes according to the strategy. This methodology helps to model and compare design alternatives, verify impacts in every evaluation criteria, and make robust and objective information-based decisions. The application of the methodology in real cases of plastic material design optimization in the automotive industry has provided remarkable results, accelerating the detection of improvement methods aligned with the strategy and maximizing the improvement in product competitiveness and sustainability. In comparison with the simultaneous application of existing mono-criteria optimization methodologies (such as “Design to Cost” or “Eco Design”) and subjective expert-based reviews, the novel methodology has a reduced workload and risks, confirming its potential for future application and further development in other polymer-based products, such as consumer goods or packaging.

## 1. Introduction

**Figure 1:**
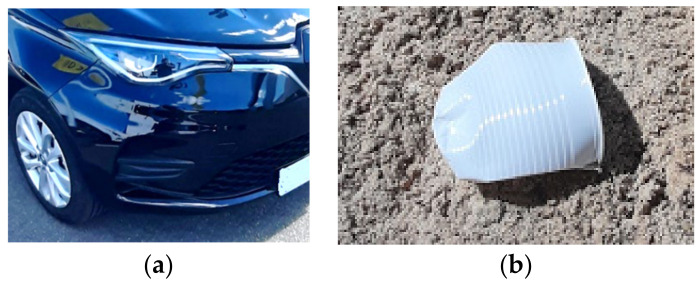
(**a**) High-quality and long-life automotive plastics, improving vehicle performance and reducing weight, fuel consumption, and emissions; (**b**) low-quality and short-life non-sustainable utilization of disposable plastics.

Plastic materials have been a revolution for many markets in previous decades, replacing other materials due to their cost competitiveness, design flexibility, light-weight or decoration and finishing properties, widening the access to convenient products and playing an important role in the development of humanity [1]. In the automotive industry, the introduction of plastic has meant a significant vehicle performance improvement and weight reduction [2,3], reducing fuel consumption and emissions, and improving the visual aspect, user ergonomics, and comfort. In the same vein, plastics offer new opportunities and trends, such as fiber-reinforced composites [4], the design of plastic parts with diverse function integration with a non-assembly target [5], and direct part manufacturing with 3D printing processes [6,7], as well as renewably sourced bio resins [8], which are examples of long-life, competitive, and sustainable plastic applications, removing the need for molds, processes, stocks, or long-distance shipping. However, on the contrary, some other plastic applications, such as disposable or short-life products, are less sustainable examples, resulting in raw material waste and sustainability problems, as graphically compared in Figure 1. This has affected the customer sustainability perception of plastics, and also forced the development of public programs to reduce the utilization of low-quality plastics.

Companies currently producing or designing plastic products must adapt as fast as possible to market requirements and opportunities, reviewing their product and process design to continuously improve and to detect optimization opportunities to improve competitiveness and sustainability. The development and utilization of specific methodologies for this transition could accelerate this adaptation and represent a key competitive advantage for success and sustainability.

Due to the intrinsic subjectivity of product design activities, some studies have attempted to understand and evaluate their designs according to attributes [9], and improve their designs with QFD and FMEA [10]. There are also several different optimization methods in automotive industry design [11,12], mainly based on functional design tools; QFD; FMEA; and methods such as Fuzzy logic, VIKOR or MCDM (multi-criteria decision making), which focus on the design phase of new products. In particular, for plastic products and related processes, widely applied strategies to check the design and process feasibility include finite elements modeling (FEM) and injection rheology simulation, as well topology review [13,14] and “concurrent design” principles [15]. Some works are also focused on characterization and design optimization specifically oriented towards new processes, such as additive manufacturing [16,17], or materials, such as fiber-reinforced plastics [18,19], and focused on plastic process knowledge capture strategies [20]. Regarding product sustainability, some works also describe material selection [21] in consideration of end-of-life recyclability [22]; the characterization of bioplastics or natural fiber-reinforced plastics for different applications [8,23,24]; and the plastic product lifecycle, end-of-life costs, and environmental impact calculation [25,26,27,28].

Regarding methods for design review and optimization, in some competitive industries, continuous improvement methods are widely applied, such as Lean, Kaizen or Six-Sigma [29,30], also combined with Fuzzy Logic and VIKOR procedures [11,31], but in this case, the main focus is on manufacturing process optimization in the serial production phase. For design optimization, mono-criteria improvement methodologies can be applied, such as “Design for Manufacturing” [32] or “Design to Cost” [33]. Some interesting works define a multi-criteria evaluation for parametric design and topology optimization [34]; in this case, we only focused on the 3D printing process. Others define an analytical hierarchy for design alternative selection during the concept design phase of automotive plastic bumpers [35]. Others have used multi-criteria decision making (MCDM) for generic material selection [36] or Fuzzy Logic [11,37,38] procedures, as well customer information mining [39] for decision support during the product design phase. However, based on the authors’ experience, it is interesting to highlight how some of these methods are diverse, and focused on individual processes or material characterization, and their related checks and validations, making the robust comparison of different alternatives a difficult task. Some of the existing methods are applicable only in several phases of the product lifecycle, making them difficult to apply in real cases. These diverse approaches also cause errors in evaluations in some cases, potentially leading the designer to a result far from a viable competitive and sustainable plastic product.

As an example, even if applying some existing plastic design methods [13,14,29,30] to the single-use plastic cup, as seen in Figure 1b, the result could be a cup with an OK result in FEM simulations, rheology, and molding feasibility, as well as a lean production process. However, designers and manufacturers need a wider view of their design, considering other aspects, such as product lifetime or end-of-life waste volume, making it possible to detect that this disposable cup is a short-life, and non-sustainable solution. Even if the cup designer or producer detects these weaknesses, there is a lack of methodologies that help to explore, compare or select design alternatives in consideration of their targets and restrictions. This is the main gap detected by authors for the development of this “Design for Target” multi-criteria design optimization methodology, but the aim of this work is quite different to all referenced methods, as it focuses on the real need for plastic industry transformation, as described in the following objectives:

- The methodology has the objective of plastic product and process design review and optimization at any product lifecycle milestone, from concept to end of life, in order to make it possible to apply in any company and lifecycle stage.
- It must be capable of capturing the business strategy, which could be different at each milestone, and must be translated into a set of improvement “target” and restrictions.
- It is focused on the “multi-criteria” evaluation of design alternatives, the detection of optimization strategies according to targets and restrictions, determining changing impacts and making objective and information-based design decisions.
- The methodology must be easily integrated as an additional “layer” that is compatible with any company size, organization, design strategy or knowledge management system, without required changes, simplifying its implementation and accelerating knowledge capture and application results.
- The methodology is expected to be applicable to “discrete events”, to simplify the early adoption of the methodology in any product lifecycle phase. It also facilitates the implementation of the methodology, as it does not require any change to the existing methodologies or organizations, in any company size or market setting.
- The methodology must support knowledge capture as a “Knowledge-Based Engineering” (KBE) tool, helping in “incremental” methodology development and a continuous improvement flow, and ensuring long-term utilization. It must be suitable for the future implementation of learning, artificial intelligence (AI) or data mining procedures.

**Figure 2:**
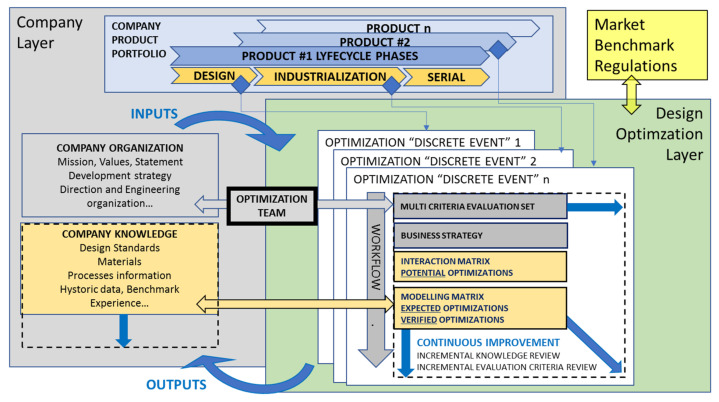
Design optimization “layer” compatible with any company size and organization, and applicable at any product lifecycle milestone. Blue arrows represent the “incremental” methodology, continuous improvement, and knowledge capture philosophy.

With these main objectives, the methodology development started with a working principle, integration and schema, as shown in Figure 2.

## 2. First Application Scope of the Methodology: Automotive Plastic Materials

**Figure 3:**
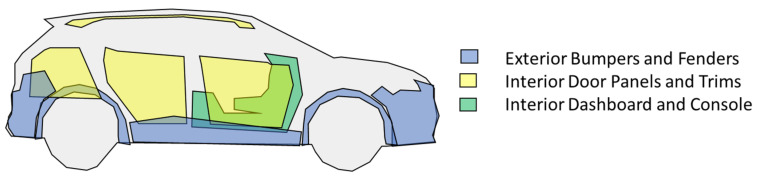
First application scope of the three main vehicle plastic materials.

The methodology was first developed to be applied in a competitive and exigent market, such as automotive plastic materials. In detail, the first application is the design review and design optimization of the three main vehicle interior and exterior plastic materials, usually representing the majority (around 58%) of the average vehicle plastic weight [4], (p. 220), as shown in Figure 3.

The application material has mainly plastic injection parts made from polypropylene, glass fiber-reinforced polypropylene, ABS, and polycarbonate materials, some of them with different aesthetics (mold texture, painting, chroming, mass tinted materials, etc.). Some other parts include additional processes, such as thermo negative or slush skin formation, skin wrapping, and polyurethane foaming, as well as system assembly, plastic welding, and gluing. The application of the methodology also makes it possible to integrate actors and expertise from different activities—from vehicle design and product engineering, to process engineering and manufacturing processes expertise, as well purchasing and quality support functions. We also considered the application in different lifecycle phases for different vehicles, business strategies, and optimization targets for each event, allowing us to test and validate the methodology in a wide range of representative situations.

## 3. Methodology Development

**Figure 4:**
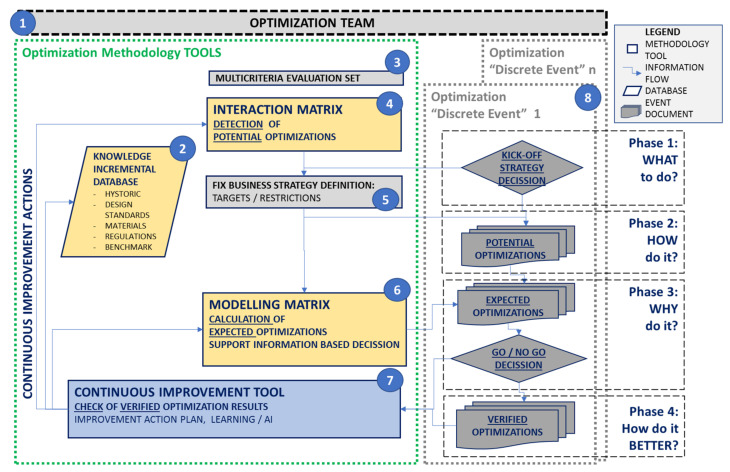
Detailed flowchart of methodology: phase and tool schema.

According to described aim and objectives, the methodology development started from a previously published basic flowchart [40], which describes the main information sources and information flow principle. Starting with this first base flowchart and considering the previously described objectives and application scope, a second detailed flowchart, which describes the main information sources, application phases, tools, and documents, was developed, as described in Figure 4.

The methodology has a set of tools, as shown on the left side of the schema in Figure 4, and a “discrete event” utilization strategy and documents, as shown on the right side of the same schema. The aim of the methodology tools is to be the knowledge capture base, which is oriented towards long-term profitability based on “incremental” development in a comprehensive and continuous improvement way [41]. The discrete event strategy and documents are defined to take maximum advantage of the methodology tools in every application loop, and to help to document the analysis, calculations, and decisions carried out in each event. The methodology tools are shown with numbers inside blue circles in Figure 4, and each of the main characteristics are described in the following:

1. Optimization team: The first step is to set up the team who will take part in the optimization process. The best strategy for this is defining a multi-disciplinary team representing all company departments and wide knowledge areas. In our application case, the stable collaboration team was 15 people with profiles covering the main company knowledge areas, cumulating near 300 years of experience; as shown in Table 1, 100% have a university degree and 33% hold a PhD. In addition, during the methodology development and application, occasionally, 12 additional people shared information or took part in decision making.
2. Knowledge “incremental” database: Once the optimization team has been identified, the first step of the methodology development was to request team members to collect related information, and disseminate it to the rest of the team, including design standards, plastic material information, process guidelines, or regulations to be respected. The “incremental” aim of the methodology enabled us to start applications in real cases, even with “partial” knowledge collection, helping to detect lacking areas and develop a comprehensive knowledge base. As an example, in the first application, we detected a lack of benchmark information, as an interesting optimization opportunity; then, an existing benchmark vehicle database [42] was included in the knowledge base.
3. Multi-criteria evaluation set: This is usually defined by the company directors, based on existing key performance indicators (KPIs), and is as complete and stable as possible to allow comprehensive and comparable evaluations. In our case, we used the QCDP set of KPIs (quality, cost, delivery delay, and performance), which is well known in the automotive and product development industries [43,44]. However, we detected that this set of criteria lacks an environmental impact evaluation [25,26,27], and as a consequence, the team developed a QCDP+E set (+E denotes environmental impact). In addition, the team detected the need for several “sub-criteria” for each main one, which would be useful for better impact evaluation, and would also enable the detailed definition of targets and restrictions, resulting in the criteria set shown in Table 2.

After the first application in real cases, the team reviewed and approved the set of evaluation criteria, and also identified “reference members” for the evaluation and continuous improvement of each criterion according to their areas of expertise.

1. The interaction matrix is the central tool of the methodology. It concentrates this information in a profitable format, organizing the following information in a matrix format:
  - The multi-criteria evaluation set was placed in the columns, as described in the previous paragraph.
  - A list of “potential design optimization” or “changes” was placed in rows, identified by the optimization team members, based on a related knowledge database and expertise, and ordered considering the origin of the opportunity (initially in three main categories: product design, process design, and market/benchmark).

**Figure 5:**
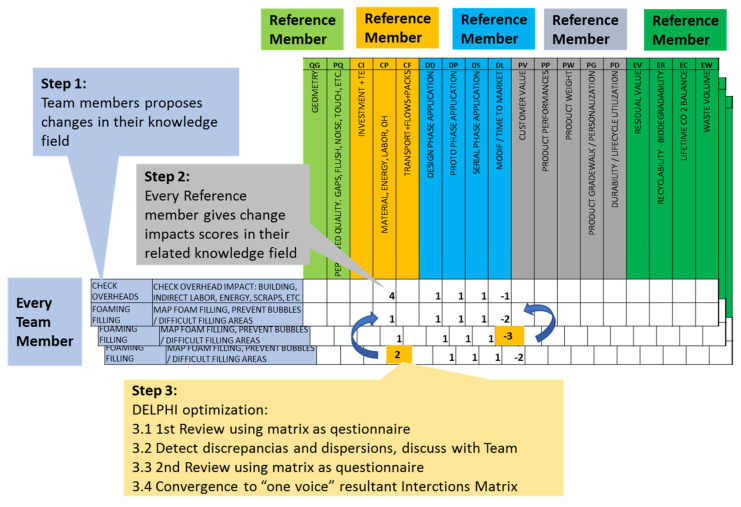
Interaction matrix development steps schema.

This interaction matrix was created in a “collaborative” manner by the optimization team, according to the three steps defined in the methodology and shown in Figure 5.

- Step 1: Each team member could propose changes in their knowledge or expertise area, mainly based on their experience, obtaining qualitative information and considering the methodology as an effective “knowledge capture” system.
- Step 2: Every change was evaluated based on its positive or negative impact on every criterion of the multi-criteria evaluation set. The author proposing the change and the reference members had to discuss every impacted criterion to reach a base quantitative value or “score” according to an agreed classification, with values from −5 to +5 (unfavorable or favorable impact, respectively), according to Table 3. The value could be also discussed with all team members, in order to ensure objectivity.
- Step 3: Once the base value for the impacts had been defined, the entire interaction matrix was evaluated by the entire team, using a Delphi or ETE (Estimate-Talk-Estimate) methodology, and the group “one-voice” opinion output was set. The Delphi method [45,46] is well known and considered an appropriate system to refine the subjective or varied knowledge of an expert team (as in our case), using questionnaire iterations, converging to the most accurate answer or solution based on collaborative knowledge. According to the Delphi rules, at least two optimization rounds are required, each one using the entire interaction matrix as a questionnaire distributed to all members of the structured optimization team (as shown in Table 1), allowing experts to anonymously review and, in our case, correct any score that they consider incorrect, resulting in a corrected matrix. The answers were collected anonymously in a shared online spreadsheet, and then evaluated by one delegated member of the team (in our case, the DELPHI “facilitator” role was performed by one of the methodology authors). The answer matrix was evaluated to verify the convergence of collective and stable answer scores by calculating the following:
  1. Arithmetic means of the answered scores, to allow reference members to evaluate their previous scores and agree to set the answer mean as the resultant score for the impact.
  2. Detection of dispersions or lack of stability in answer scores based on normal distribution calculation. In this case, disperse values allowed the team to detect the need for the review of the scores and changes to clarify the origin of these dispersions (usually misunderstandings around changes or evaluation), better explain them to the team, and then re-review the scores to obtain the agreed resultant score for the dispersive values.

After these three steps, and with all these resultant scores, it was possible to build a reviewed interaction matrix, which was the result of the team’s “one voice” and was useful for application in the following stages. It must be highlighted how easily the process could obtain the “one voice” interaction matrix, based on the experts’ knowledge capture and review strategy.

**Figure 6:**
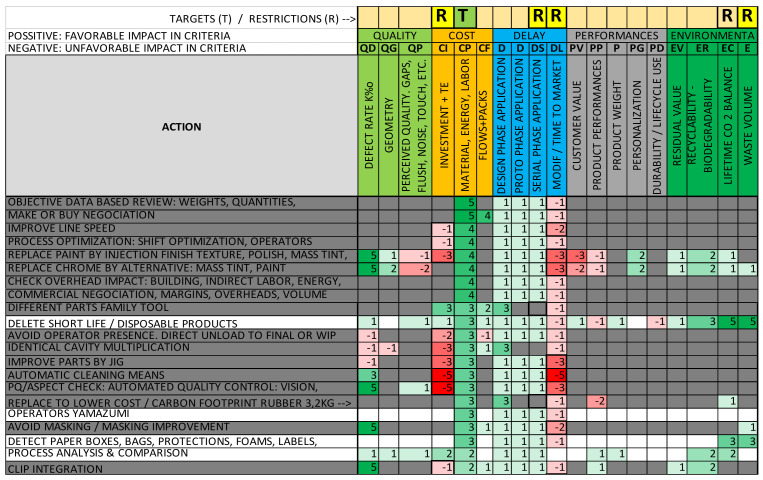
Interaction matrix example: columns: application of “target” (T) and “restrictions” (R); rows: potential optimizations; those not respecting restrictions are filled in in grey (partially shown).

The resultant interaction matrix in our application case is (partially) shown in Figure 6, and the full version is shown in Appendix A, with the multi-criteria evaluation set in columns, the potential optimization changes in rows and their impact in every evaluation criterion shown as scores in the rows, as described previously.

2. Business strategy definition: Once the interaction matrix had been defined, the next step of the methodology was the tool that allowed the target of the optimization to be fixed according to the company business strategy. This strategy is usually fixed by the company directors and is frequently different at every moment or project milestone. As an example, in the development phase, the key strategy target could be “respect planning and budget”, and in the serial production phase, the target could be “maximizing cost reduction actions, with as less investment as possible”. This strategy is usually fixed by “verbal” qualitative information, and the key is “translating” this verbal information into parametric and quantitative information.

The methodology enabled the “translation” of a “verbal” strategy into a set of quantitative “Targets” and “Restrictions” in the evaluation criteria set defined in Table 2. To this end, the most common logical and relational operators were used (=, ≠, >, <, ≥, ≤, NOT ¬, OR ∨, AND ∧, IF: THEN: ELSE), which were applied to every criterion or sub-criterion of the multi-criteria set (with their acronym from Table 2 shown in parentheses in the following sample) to enable the classification of design changes and calculate the priority for each change based on its positive or negative impact on each criterion and sub-criterion and in respect of restrictions.

To explain the “translation” process, the next example is based on a real application case, with a relative complex verbal strategy:

- Verbal strategy: “During serial production phase, detect cost reduction actions, with no investment, with application delay less than 1 month, and not affecting negatively lifecycle CO_2_ emissions or waste volume”.
- Translation into quantitative “Target” and “Restrictions” to criteria set:
  → Target: Detect and order changes by favorable impact in the “Production and Manufacturing Cost Reduction” sub-criteria = (CP) ≤ 1
  → Restrictions:
    1. Applicable in serial phase = (DS) ≥ 1;
    2. Investment or entry ticket = (CI) ≤ 1;
    3. Impact on delivery time/planning = (DL) ≥ −1;
    4. Impact on lifecycle CO_2_ emissions = (EC) ≥ 0;
    5. Impact on waste volume = (EW) ≥ 0.

With the translation of the strategy into a set of target and restrictions, it was possible to define an algorithm to search the “interaction matrix” and detect and classify the changes that met the defined strategy, as shown below, in pseudocode, with comments after the # symbol.

→ Result = [Interaction Matrix: 1→n]; #run through matrix lines
  IF: CP ≤ 1; AND → #verify accomplishment of target.
  IF: DS = 1; AND → #verify accomplishment of restriction 1.
  IF CI ≤ 0; AND → #verify accomplishment of restriction 2.
  IF DL ≥ −1; AND → #verify accomplishment of restriction 3.
  IF EC ≥ 0; AND → #verify accomplishment of restriction 4.
  IF EW ≥ 0; THEN → #verify accomplishment of restriction 5.
  SORT: CP (Max→min) → #classify result according to target.

It is possible to apply this kind of algorithm to any mathematic software working with a matrix or also any database or spreadsheet software that can filter and sort information. In the practical application, the lines of the interaction matrix were verified for “target” and “restrictions”. Lines that did not meet these requirements were rejected. As shown in Figure 6, changes were ordered based on their impact on the target.

This procedure result was an ordered list of the best “POTENTIAL Optimizations” in the interaction matrix, which respected the business strategy. This allowed profitable work in the following stages: accelerating design improvement and improving work effectiveness in comparison to existing expert subjective optimization searches; and accelerating changes to meet strategy, market, and customer needs.

**Figure 7:**
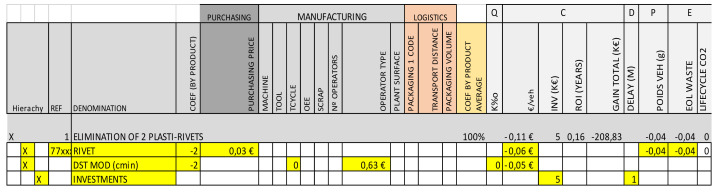
“Modeling Matrix” with detailed impacts calculation (partially shown).

3. Modeling matrix: Once the “POTENTIAL Optimizations” had been detected and listed according to the strategy, the next step was helping the team to verify if these changes were fully applicable to a real case and helping to perform the robust impact modeling and calculation of the “EXPECTED Impacts”, which enabled the team and management to make Go/No Go decisions around changes based on clear and robust information of the impact calculation in all multi-criteria sets, as shown in Figure 5. The structure of the modeling matrix helped the team to model every optimization impact considering all stages of the product lifecycle: design, manufacturing, product utilization, and end of life. In order to speed up the calculation, the impacts were modeled in a “variational” way, accurately modeling all changes from the baseline in an efficient manner, as shown in Figure 7.

This tool enabled the addition and modeling of any subjective origin optimization, including modeling as an additional method to capture knowledge and continuously improve the methodology.

4. Continuous improvement tool: The methodology has an “incremental” orientation, which allows it to be applied from the first stages of the knowledge capture process, but it also needs a tool to ensure continuous improvement. This tool performs the following tasks:
  1. Checks the verified impacts of every Go decision, in order to detect any deviations in previous stages (potential and expected impacts).
  2. Captures any subjective origin actions and checks real results in the same way.
  3. Collects and captures any new information used during the process: benchmark good practices, regulation changes, knowledge review proposals, etc.
  4. Builds an “Improvement Action List” based on this information.
  5. Periodically performs a methodology review with the optimization team to review listed actions and, in our case, correct scores of the Delphi validation, as previously described, and update the knowledge base and methodology tools.

These tool and actions ensured the periodical review and optimization of the tools of the methodology as a knowledge capture system, with possible development to some automatic detection of deviations and new information, as well as an artificial intelligence application.

5. Methodology application in discrete events: The steps of a typical application in a discrete optimization event, once the main tools of the methodology have been developed, as shown in Figure 4, are described in the following:
  1. Phase 1: What to do? Kick-off the event, define the team members, extend team collaborators, and optimize the scope. The company directors provide the business strategy for the event, usually in verbal format.
  2. Phase 2: How to do it? The team performs the translation into the quantitative “target” and “Restrictions”, applying it to the most updated interaction matrix, and obtaining the list of potential optimizations. The team adds any additional subjective-origin proposal. The resulting optimization list feeds into the modeling matrix for the event.
  3. Phase 3: Why do it? The team collects the information and runs calculations until the modeling matrix is complete with detailed calculations of the expected impacts of the optimization list, providing clear and robust information to help directors make decisions regarding the Go-NoGo of every change.
  4. Phase 4: How to do it better? The team tracks the application of Go decisions and calculates the verified impacts once changes are applied. The team compares verified impacts with previous phases of potential and expected impacts, and in the case of deviations, develops an improvement action list. If any other deviation or useful information is found during an event, the action list is also evaluated by the team after every optimization event.

## 4. Integration of Historical Database Information

As mentioned above, the aim of the methodology is to allow application from early stages, and based on knowledge capture, to perform “incremental” development. In our case, one clear opportunity for the development of the interaction matrix was the integration of historical database information. The team detected available databases regarding product modification and problem-solving events from several different companies, plants, and vehicles. These kinds of databases are useful in our case, as a source of historical information to review the interaction matrix, in particular, to review change impact scores in application delays, cost reduction opportunities, and impacts on quality evaluation criteria. All collected databases used formats and codes based on automotive standards, and were relatively easy to compile; then, the statistical software Minitab v17 application was analyzed as follows:

### 4.1. Cost Optimization Modification Database

Information from cost reduction actions applied in the period 2019–2021 and derived from 27 different plants worldwide. The database has more than 2400 lines, and 1080 modifications directly related to plastic materials were filtered: type classification, as shown in Table 4, and the information on each line, as shown in Table 5.

With his information, a statistical analysis was performed to increase the precision of the interaction matrix impact scores.

ANOVA analysis of saving as a quantitative variable versus type as an independent/qualitative variable/factor was conducted to obtain information surrounding the potential of each optimization type, based on statistical confidence intervals. The results are shown in Table 6. With an adequate significance level, it is possible to determine the highest potential in diversity reduction, make-or-buy, and logistic flow-related actions.

ANOVA 1-factor analysis of delay as a quantitative variable versus type as an independent/qualitative variable/factor was performed to obtain information surrounding the application delays of each optimization type. With results, as shown in Table 7, with adequate significance level, it is possible to determine the longest application delays for make-or-buy actions, and the shortest delays for packaging- and process-related actions.

### 4.2. Quality Problem-Solving Actions and QRQC Database

A database with approximately 4500 lines for problem solving or QRQC (Quick Response to Quality Concern) actions was applied in several different vehicles and companies. Then, 694 actions directly related to vehicle plastic materials were filtered. These actions were also classified considering the type of quality issue according to Table 8. Each action has information regarding the type of problem or defect, defect gravity, and solution delay, as shown in Table 9.

With this information size, statistical analysis was conducted to review the interaction matrix scores for impacts in duality criteria, as described in the following paragraphs.

ANOVA analysis of solving delay as a quantitative variable versus defect type as an independent/qualitative variable/factor was performed in order to detect the root causes with the most complex solution and longer solution delay/impact, and the results are shown in Table 10; with a sufficient significance level, it is possible to determine the longest delays for PF (process/means) and CG (conception/geometry) defect types.

All results of the described historical database analysis were incorporated into the methodology applying the continuous improvement tool, sharing the results with the team to update the interaction matrix tool with this information, and applying it to cells highlighted with blue borders, as well as validating it with an additional Delphi boucle, and the resultant cell values are shown by blue lines in the interaction matrix.

It is interesting to highlight that it is still possible to improve the methodology with additional analysis of the existing data, or collecting additional databases from different sources, with the application of “Big Data” principles, or any other information source. The “incremental” aim of the methodology and the continuous improvement tool is to help to capture knowledge benefit from this information. The resultant interaction matrix currently in use is shown in Appendix A. It contains 123 potential optimization changes (in rows), classified as described in Table 11. For each change, there are impact scores in the 19 multi-criteria evaluation set of criteria and sub-criteria (in columns), providing around 2300 interactions, with 1200 already valued (52%) and approximately 280 (12%) with additional validation based on historical information, resulting in a knowledge “one voice” validated by the team for cooperative design optimization research.

## 5. Methodology Application in Real Cases and Results

**Figure 8:**
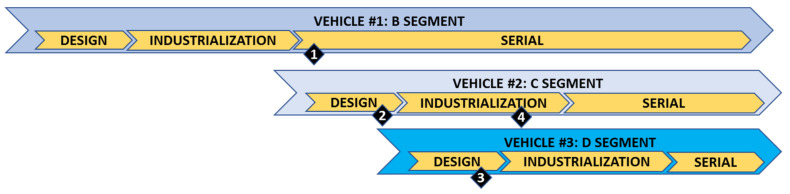
First applications of methodology in different vehicles, segments, and stages.

The main objective of the methodology development was to improve the design optimization duties, reduce subjectivity, accelerate optimization, and improve the knowledge capture, ensuring the consideration of the company directors’ business strategy and customer and market demands. The methodology was already applied during 2020 and 2021 in four “Discrete Optimization Events” for three different vehicles of the European market; in three different market segments (B, C, and D); and in different vehicle lifecycle milestones, from the design phase to industrialization and serial production phases, as described in Figure 8.

As expected, each optimization event had different scopes and strategies, with different teams (with several “shared” team members). This helped to test the methodology in different scenarios, which is the best way to validate it. The four event strategies and results are listed in detail in Table 12.

In order to analyze the effectiveness of these first methodology application events versus the existing expert team’s subjective optimization research in the same events, a comparison of objective evaluation indicators was performed, as shown in Table 13.

These results confirm the effectiveness of the new methodology in comparison to the existing expert team’s subjective optimization research applied in the same period:

- Higher successful proposal application ratio.
- More effective change detection member/workload ratio.
- Accurately estimated impact modeling, and precise information provided to support management decision making, thus reducing risks.
- Higher verified results and better workload/results ratio, including the workload for methodology development.

The higher success ratio of proposals and better methodology performance in comparison to previously existing subjective-based research are probably due to the innovative business strategy translation into targets and restrictions, which makes it much easier to detect most potential optimizations to be studied, and helps to model the alternatives with detailed impacts in the multi-criteria evaluation set, providing robust information to support decision making. In addition, the robustness of the methodology development, based on knowledge capture and historical data sources, is important.

The current limitations of the methodology seem to be related to the historical database used, although the massive data checked only partial interaction matrix scores. It is likely that information will still need to be collected in order to increase statistical analysis and information robustness. Another limitation is related to the continuous improvement. Although we started with a limited knowledge database, which could have affected the results of the first applications, the first utilization of the methodology confirmed its potential for lacking knowledge detection and knowledge capture simplicity, rapidly increasing knowledge robustness, but reducing the effectiveness of the first optimization events.

## 6. Conclusions

The development of the methodology enabled the collection of useful plastic product and process design-related knowledge that was previously varied and based on subjective experience. Knowledge collection was performed in a “natural” way and in a team workload-efficient manner. The construction of the interaction matrix based on this knowledge and the statistical analysis of historical data, reviewed and validated as the expert team’s “one voice”, provided considerable robustness to ensure confident application in real cases, allowing easier understanding and application and reducing subjectivity, representing a powerful tool to collect company knowledge and accelerate transfer and learning.

The application of the methodology in real cases in a market as competitive as automotive plastic materials has also provided good results from the early development stages, allowing the team to carry out “incremental” development, detect more optimization changes, model the impacts accurately and provide precise information for decision making. The defined information structure and continuous methodology improvement strategy also enable long-term updates and utilization. It is important to highlight that it has already been used for more than one and a half years, with several successful reviews and optimization/completion loops.

Therefore, it is possible to conclude that the developed methodology demonstrated an advantage in comparison to existing subjective optimization research processes. The team members and directors involved in application have provided favorable opinions regarding the effectiveness and effort–results ratio, the robustness of information during the optimization process and the verified results of the application in real cases.

In the near future, the methodology will be applied to additional design optimization events to increase its accuracy and effectivity. The application also allows the methodology and utilization experience to be shared with more teams and stakeholders, as a first step for diffusion into potentially interested departments and stakeholders inside the authors’ companies, who have already confirmed their interest in propagating the utilization of the methodology. The authors are also considering the development of training, support materials, and a comprehensive interface, probably as a web-based application that guides and supports users from stand-alone methodology applications into real cases, helping to expand the application into different plastic markets and companies, which maximizes its competitiveness and sustainability impacts. We also expect to further develop the methodology with continuous improvement strategies via the incorporation of additional information databases, related analysis, and periodical knowledge-based reviews to incorporate any new trend, benchmark solution, process, or material.

The wide application of this kind of methodology, which is specifically oriented towards accelerating the product and process design optimization of plastic products, enabling plastic product producers to accelerate this adaptation, is the only way to ensure competitiveness, long-term sustainability, and environmental impact reduction in plastic products.

## Figures and Tables

**Table 1:** Optimization team members: expertise area and experience.

Member	Expertise Area	Experience (Years)
1, 2P	Plastic Product Design and Development	18, 16
3, 4P	Product Management, Design, and Marketing	22, 18
5, 6	Injection Process Engineering and Quality	16, 24
7, 8	Paint and Assembly Engineering and Quality	12, 16
9	Tooling Engineering	24
10, 11, 12	Costing / Finance/Monozukuri Management	30, 24, 18
13P, 14P	Materials Experts	30, 8
15P	Environmental Impact Engineering	12

**Table 2:** Table of current main QCDP + E criteria, sub-criteria, and their codes.

Main Criteria	Sub-Criteria	Code	Reference Members
Quality	Quality Defect Rate K ‰ (K/1000 parts)	QD	5,6,7,8
	Geometry	QG	
	Perceived Quality	QP	
Cost	Investment and Entry Ticket	CI	9,10,11,2
	Production and Manufacturing Cost	CP	
	Flows and Transport Cost	CF	
Delivery and Delay	Applicable in Design Phase	DD	1,2
	Applicable in Prototype Phase	DP	
	Applicable in Serial Phase	DS	
	Impact in Delivery Time/Planning	DL	
Product Performance	Product Customer Recognized Value	PV	3,4
	Product Performances	PP	
	Product Weight	PW	
	Product Grade Walk/Personalization	PG	
	Product Durability	PD	
Environmental Impact	Residual Value and Residual Recuperation	EV	13,14,15
	Recyclability	ER	
	Lifecycle CO_2_ Emissions Balance	EC	
	Residual Waste Volume	EW	

**Table 3:** Classification for each change impacts “scores” in every criterion of multi-criteria evaluation set.

Impact	Favorable					Neutral	Unfavorable				
Value	5	4	3	2	1	0	−1	−2	−3	−4	−5

**Table 4:** Classification types for cost reduction modification database.

Type Code	Optimization Source	Quantity
LS	Logistic flows	27
SR-DM	Materials	199
SR-DS	Product Diversity Reduction	10
SR-L	Packaging	52
SR-MB	Make-or-Buy/Production Relocation	115
SR-PL	Process Optimization	45
SR-R	Product Redesign	199
SR-RC	Product Right Content	207
SR-RS	Product Right Sizing	214
SR-SA	Negotiation	12

**Table 5:** Information for each cost reduction modification in database.

Cost Optimization Changes (1080)	SAVING (EUR/Veh)	APPLICATION DELAY (days)	TYPE (10)

**Table 6:** ANOVA results for saving versus type.

Type Code	Qty.	Mean	Std. Dev.	95% Conf. Interval
LS	27	−1.90	3.365	(−2.541; −0.839)
SR-DM	199	−0.536	0.799	(−0.849; −0.222)
SR-DS	10	−3.130	5.770	(−4.530; −1.730)
SR-L	52	−0.733	1.522	(−1.346; −0.120)
SR-MB	115	−1.940	3.623	(−2.353; −1.528)
SR-PL	45	−0.601	1.283	(−1.260; 0.058)
SR-R	199	−0.749	1.328	(−1.063; −0.436)
SR-RC	207	−1.344	2.595	(−1.651; −1.037)
SR-RS	214	−0.986	2.429	(−1.288; −0.683)
SR-SA	12	−1.663	1.717	(−2.939; −0.387)
Std. Dev. Grouped = 2.253; F Value = 5.550; *p* Value ≤ 0.001				
95% of sample confidence intervals and residuals. 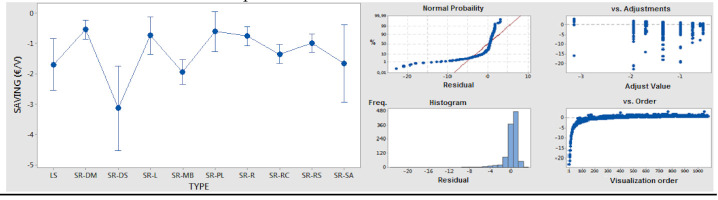				

**Table 7:** ANOVA results for delay versus type.

Type Code	Qty.	Mean	Std. Dev.	95% Conf. Interval
LS	27	279.0	193.0	(201.3; 356,8)
SR-DM	199	241.7	244.7	(213.1; 270.4)
SR-DS	10	197.5	122.7	(69.7; 325.3)
SR-L	52	103.0	125.8	(47.0; 159.1)
SR-MB	115	391.9	251.6	(354.2; 429.6)
SR-PL	45	105.8	153.2	(45.6; 166.0)
SR-R	199	244.1	192.6	(215.4; 272.7)
SR-RC	207	265.4	198.8	(237.3; 293.5)
SR-RS	214	241.3	185.7	(213.7; 269.0)
SR-SA	12	244.0	219.7	(127.3; 360.7)
Std. Dev. Grouped = 205.950; F Value = 11.820; *p* Value ≤ 0.001				
95% of sample confidence intervals and residuals. 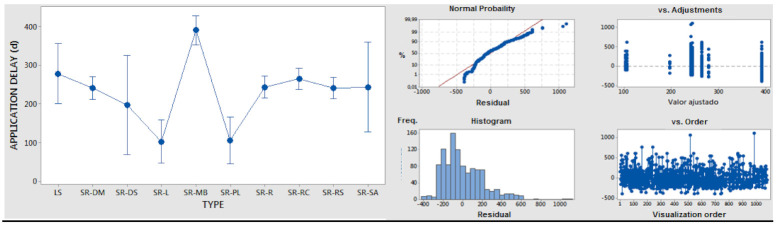				

**Table 8:** Defect type description of quality actions database.

Defect Type	Description	Quantity
CC	Conception/Clippings	96
CG	Conception/Geometry	24
CP	Conception/Performances	5
CR	Conception/Noise	14
EC	Electric Connectors failure	31
EE	Electric Equipment failure	34
PA	Process/Aggression	94
PF	Process/Manufacturing defect	37
PI	Process/Injection defects	15
PM	Process/Assembly defects	133
PY	Process/PokaYoke error	107
QP	Quality/Perception	100
QA	Quality/Aspect	4
	TOTAL	694

**Table 9:** Information for each quality action in database.

Quality Actions (694)	SOLVING DELAY (days)	DEFECT TYPE (13)

**Table 10:** ANOVA results for solving delay versus defect type.

Defect Type	Qty.	Mean	Std. Dev.	CI 95%
CC	96	125.3	91.2	(106.5; 144.0)
CG	24	154.5	125.8	(112.1; 196.9)
CP	5	87.7	95.4	(5.2; 170.4)
CR	14	94.9	89.6	(45.5; 144.2)
EC	31	105.6	85.0	(72.5; 138.8)
EE	34	108.3	87.0	(76.1; 140.5)
PA	94	124.0	89.6	(105.0; 142.95)
PF	37	171.4	117.4	(141.0; 201.7)
PI	15	83.9	57.6	(36.2; 131.6)
PM	133	110.6	102.2	(94.5; 126.6)
PY	107	124.9	88.7	(107.0; 142.8)
QP	100	112.8	86.5	(94.2; 131.3)
QA	4	90.8	51.9	(−1.7; 183.2)
Std. Dev. Grouped = 94.120; F Value= 1.80; *p* Value= 0.044 95% of sample confidence intervals and residuals.				
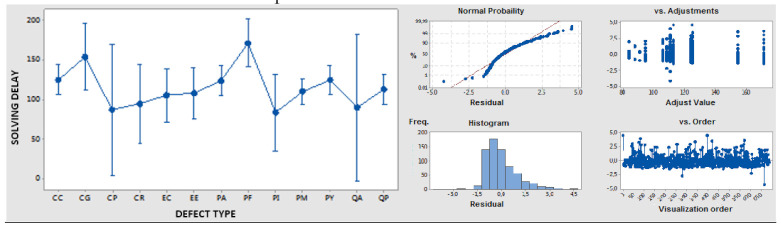				

**Table 11:** Classification of current interaction matrix changes.

Hierarchy 1	Hierarchy 2	Quantity
Product Design	Functional Design Topological Design Material Selection	58
Process Design	Tooling Design Process Design Flows Design	37
Market Analysis	Supply Chain Analysis Benchmark Analysis	28

**Table 12:** First 4 applications events, strategies, and results of the methodology.

Event	Phase	“Verbal“ Strategy	Results
1	Serial	“Cost reduction, minimum Investment, less than 3-month Application Delay”	21 Actions proposed 14 Changes applied −16.5% Cost reduction
2	Design	Design Phase. “Design to Cost reduction target, ROI less 6 months. Respect project Planning”	14 Actions proposed 11 Changes applied −16.2% Cost reduction
3	Design	“Design for Quality: Detect Quality potential risks, propose alternatives, respect project planning”	31 Actions proposed 28 Changes applied −47% Potential Defect rate (K‰)
4	Industrialization	“Right Content Benchmark analysis, detect cost and product diversity optimization opportunities”	16 Actions proposed 12 Changes applied −10.5% Cost reduction

**Table 13:** Results comparison between methodology and experts’ team subjective research.

Result	Expert Team Subjective Optimization Research	Optimization Methodology Application
Proposal success ratio (%)	48/124 = 39%	65/82 = 79% (+102%)
Success proposals/member (Qty.)	19	65 (+242%)
Cost saving/member (EUR)	EUR 28.65	EUR 49.4 (+72%)
Workload/success proposal (h)	23.6 h	13.6 h (−42%) (Including methodology development)

## Data Availability

All the data used to support the findings of this study are included in this article.

## Appendix A

Appendix A includes the current status of the interaction matrix.
